# Chemotherapy- and cancer-related nausea and vomiting

**DOI:** 10.3747/co.2008.171

**Published:** 2008-01

**Authors:** D.G. Warr

**Keywords:** Nausea, vomiting, emesis, chemotherapy, 5-hydroxy-tryptamine_3_, aprepitant, neurokinin

## Abstract

Approximately one half of cancer patients will experience nausea or vomiting during the course of their disease either because of the cancer itself or because of their treatment. Emesis attributable to cancer warrants a careful investigation to determine whether a treatable underlying cause is responsible. Interventions using dexamethasone and octreotide may reduce vomiting attributable to bowel obstruction. In the absence of a bowel obstruction or a correctable cause, the usual approach is a sequential trial of antiemetics guided by considerations of cost and side effects.

Major progress in managing chemotherapy-induced emesis followed from the use of a combination of a corticosteroid and 5-hydroxytryptamine_3_ (5-HT_3_) receptor antagonist for moderately to highly emetogenic chemotherapy. Nevertheless, vomiting still occurred in approximately 40% of women receiving chemotherapy containing an anthracycline plus cyclophosphamide and in approximately 50% of patients receiving high-dose cisplatin. The addition of aprepitant, a neurokinin 1 receptor antagonist, improved control of emesis by a further 15%–20%, and that agent is now recommended as part of standard antiemetic therapy for patients at high risk of emesis. Based largely on anecdotal experience, cannabinoids and olanzapine are sometimes also recommended in patients with refractory emesis. Phase iii trials are required to confirm their efficacy as add-ons to a corticosteroid, a 5-HT_3_ receptor antagonist, and possibly aprepitant.

## 1. INTRODUCTION

Nausea and vomiting are common problems in cancer patients throughout the trajectory of their illness. Whether these patients are receiving high-dose cisplatin 1 with the best available antiemetic therapy or are experiencing the advanced stages of cancer 2, approximately one half will experience nausea or vomiting, or both. The causes of these distressing symptoms are diverse, and they include medication, radiation therapy, and the effect of the cancer itself. Table I lists some examples. The present article reviews the approach to emesis attributable to cancer or chemotherapy.

## 2. DISCUSSION

Although nausea and vomiting are closely related, some patients experience one symptom without the other. For example, mild-to-moderate nausea is often not accompanied by retching or vomiting. On the other hand, some patients with brain metastases or esophageal obstruction report vomiting without prior nausea.

The physiology of nausea is not well understood 3. In choosing a pharmacologic approach, no distinction is usually made between vomiting and nausea; however, the literature suggests that it is easier to eliminate vomiting than nausea 4,5.

### 2.1 Cancer-Induced Emesis

The first step in the approach to cancer-induced emesis is to establish whether a remediable cause is present. A careful history, physical examination, laboratory tests, and (sometimes) imaging are required (Table II). How often a diagnosis can be made of the underlying cause for emesis is unclear.

A prospective study of 121 patients determined that 50% were experiencing nausea or vomiting or both on admission to a hospice^5^. The causes were diagnosed as “chemical” (metabolic, drug-related, infectious) in 33%, impaired gastric emptying (tumour, hepatomegaly, drug-related, ascites, other) in 44%, visceral or serosal (bowel obstruction, other) in 31%, intracranial in 8%, and anxiety in 7%. For each cause, the authors had defined, in advance, a pharmacologic approach that included one or more of haloperidol, metoclopramide, cyclizine, dexamethasone, or a benzodiazepine. Indeterminate causes were treated with levomepromazine.

Although this prospective study reported control of vomiting in 89% of patients, several problems arise in applying the results to practise. The criteria for diagnosing the causes are not listed, and no “gold standard” exists to determine the accuracy of the diagnoses. Of the study patients, 50% were already on an “appropriate” antiemetic at the time of admission, raising questions about whether the recommended pharmacologic approach was actually responsible for the improvement. After 1 week, 48% of patients had dropped out of the study. The article also did not state how often a correctable underlying cause—for example, hypercalcemia—was found.

A valid pharmacologic approach to the problem of nausea and vomiting requires a good evidence base. Unfortunately, very few controlled trials on this subject have been conducted. A systematic review of the efficacy of antiemetics in treating nausea in advanced cancer was able to find only seven randomized clinical trials: two for bowel obstruction, one for opioid-related nausea, and three in which several causes (such as bowel obstruction, brain metastases, metabolic disturbances, and medications) had been excluded^6^. The authors concluded that

- for bowel obstruction, corticosteroids were superior to placebo in one study. (The other study had a sample size that was inadequate for drawing conclusions.)
- for opioid-related nausea, ondansetron, metoclopramide, and placebo are not significantly different.
- for a non-specific cause for nausea, the 5-hydroxy-tryptamine_3_ (5-HT_3_) receptor antagonist tropisetron is superior to the more conventionally prescribed metoclopramide or chlorpromazine.

These conclusions were weakened by small sample sizes and lack of a double-blind design in some of the analyzed trials.

The approach to cancer-induced nausea and vomiting therefore remains largely empirical:

- When feasible, correct an underlying cause such as severe hyponatremia.
- In the case of bowel obstruction, use dexamethasone 7 or octreotide 8 alone or in combination (commonly accompanied by a period of “bowel rest” that relies on hydration by the intravenous or subcutaneous route) until evidence of obstruction improves.

For other causes of emesis, empiric trials of antiemetics (Table III) remain the only source of guidance. Antiemetics are typically chosen based on considerations of past practice, cost, and adverse effects. Phenothiazines or a butyrophenone, substituted benzamides, and possibly antihistamines are tried before 5-HT_3_ receptor antagonists or cannabinoids. The use of aprepitant in this setting has not been reported.

### 2.2 Chemotherapy-Induced Nausea and Vomiting

Before the 1980s, chemotherapy-induced vomiting occurred in a preponderance of patients who received cisplatin or doxorubicin. In the 1990s, an antiemetic combination of a corticosteroid and a 5-HT_3_ receptor antagonist became common practice for many cyto-toxic regimens. By the early part of that decade, that antiemetic combination had had a noticeable impact on admissions to hospital for control of emesis, leading to cost savings 9. But despite that progress, substantial problems with nausea and vomiting remained.

In two large randomized trials, 50% of patients receiving high-dose cisplatin still experienced vomiting and 58% experienced nausea in the face of standard antiemetic therapy 1. Although traditionally regarded as “moderately emetogenic,” anthracycline and cyclophosphamide chemotherapy for breast cancer evoked vomiting in 41% of patients and nausea in 67% following ondansetron and dexamethasone 10. Those outcomes were vastly better than the outcomes seen in the 1980s, but considerable room for improvement remained.

Several large comparative studies showed no difference in efficacy between ondansetron, granisetron, and dolasetron 11,12. The prevailing belief was that all 5-HT_3_ receptor antagonists were equivalent in efficacy and in side effects when delivered in the recommended doses. That paradigm was challenged by palonosetron, an intravenous 5-HT_3_ receptor antagonist with a sufficiently long half life that a single administration was sufficient 13.

In patients who received moderately emetogenic chemotherapy, two large randomized trials showed superiority for palonosetron over ondansetron 14 and dolasetron 15. In contrast, palonosetron appeared to be equivalent to ondansetron in patients who received high-dose cisplatin 16. Despite those results, the American Society of Clinical Oncology (asco) guide- lines do not recognize palonosetron as the 5-HT_3_ receptor antagonist of choice, because the therapies used in the study comparator arms were not regarded as best standard therapy 17. The true role for palonosetron will be confidently established only by randomized trials in which the standard therapy conforms to recommended practice.

Also in the 1990s, a new of class of antiemetics was discovered: the neurokinin 1 (NK_1_) receptor antagonists 18 . The NK_1_ receptor has substance P as its natural ligand and is present both peripherally and centrally. Aprepitant is the only example of the NK_1_ antagonist class that has proceeded through phase iii testing.

Aprepitant is commercially available in many countries around the world. Its efficacy has been evaluated in four phase iii double-blind randomized studies—three with high-dose cisplatin 19–21 and one with chemotherapy containing an anthracycline plus cyclophosphamide for breast cancer 10. The standard therapy arms contained ondansetron and dexamethasone; in the experimental arm, aprepitant in the currently approved dose and schedule was added to ondansetron and dexamethasone. All trials reported primary endpoints that were statistically significantly superior in the group receiving aprepitant (Table IV). A complete response was defined as an absence of retching or vomiting and no use of an “as-needed” antiemetic.

The cisplatin studies showed a 14%–20% absolute difference in complete response when aprepitant was added; in moderately emetogenic chemotherapy, the difference was only 9%. The apparently lesser improvement with the addition of an NK_1_ receptor antagonist to chemotherapy containing anthracycline plus cyclophosphamide was further explored by looking at nausea separately from vomiting or retching. Aprepitant had no detectable effect on nausea attributable to moderately emetogenic chemotherapy 10, but a statistically significant improvement was observed in the pivotal cisplatin studies 1. In contrast, for vomiting or retching alone, the superiority in the aprepitant groups was similar across the phase iii studies: a 17% difference in the moderately emetogenic trial and a 14.3%-to-22.7% difference in the cisplatin trials (Table IV). Thus, the improved control of vomiting provided by aprepitant was approximately the same whether the chemotherapy was high-dose cisplatin or cyclophosphamide and doxorubicin; however, the beneficial effect of aprepitant on nausea seemed to be limited to settings involving high-dose cisplatin. The reason for the difference is not known.

Because aprepitant is a moderate inhibitor of the cytochrome P450 enzyme (CYP3A4), concern initially arose about whether aprepitant might affect the clearance of taxanes and vinca alkaloids that are metabolized by that enzyme. Adverse effects in the aprepitant-containing arms of the phase iii trials appeared very similar to those seen in standard therapy, with no trends across the studies even though patients commonly received concomitant taxanes, vinorelbine, or etoposide 1,10. Subsequent pharmacokinetic studies that showed no effect of aprepitant on the clearance of docetaxel 22 or vinorelbine 23 were consistent with the foregoing observations.

Although no interaction with intravenous medications has thus far been demonstrated, aprepitant may have clinically relevant interactions with oral agents that have an extensive first-pass metabolism connected with CYP3A4 in the bowel wall. For example, dexamethasone is metabolized by CYP3A4, and co-administration with aprepitant results in a doubling of the area under the curve of the corticosteroid^24.^ For that reason, the phase iii clinical trials were designed to deliver approximately one half the dose of dexamethasone in the group that received aprepitant. Aprepitant would also be expected to temporarily increase the blood levels of other oral agents metabolized by CYP3A4—as do ketoconazole, erythromycin, diltiazem, and a number of other commonly prescribed medications. Through induction of enzymes, aprepitant reduces warfarin levels by approximately one third 25, and the aprepitant product monograph indicates that aprepitant may reduce the efficacy of the birth control pill. Because aprepitant itself is metabolized by CYP3A4, strong inducers of that enzyme such as rifampin and phenytoin may lower blood levels of the drug sufficiently to make it ineffective.

Aprepitant is an oral medication. It supplied as a 3-day pack: 125 mg on day 1, and 80 mg on each of days 2 and 3. Approval is being sought for a single intravenous dose of a prodrug that yields an area under the curve similar to that seen with oral aprepitant.

Standard antiemetic therapy recommended by asco 17 and the Multinational Association for Supportive Care in Cancer 26 now includes aprepitant for chemotherapy with cisplatin and with anthracycline plus cyclophosphamide. Still, several questions remain about aprepitant use:

- What should be done for patients taking other emetogenic chemotherapy such as carboplatin or ifosfamide?
- What should the aprepitant dosing schedule be for multiple-day cisplatin?
- Does aprepitant have a role as a second-line antiemetic?

Aprepitant is an important step forward, but some patients still vomit or experience substantial nausea despite its use. In addition, aprepitant is not available in some countries, and like the 5-HT_3_ receptor antagonists, it is relatively expensive and may therefore not be an option for some patients. Other agents that might be added include a dopamine receptor antagonist (for example, prochlorperazine), a cannabinoid (nabilone or dronabinol), and the atypical antipsychotic olanzapine. Prochlorperazine appears to have very modest effects on chemotherapy-induced nausea and vomiting 27. Cannabinoids have some anecdotal support, but the data from clinical trials antedate the introduction of 5-HT_3_ receptor antagonists, and in recommended doses, significant problems with sedation and dysphoria arise. Olanzapine blocks multiple receptors, including dopamine receptors 28. Anecdotal accounts and phase ii studies suggest an important antiemetic effect 29,30, but phase iii data are not yet available.

Tables V and VI outline a reasonable approach to antiemetic therapy that is consistent with the available evidence.

## 3. SUMMARY

Cancer-related nausea results from a wide variety of causes, some of which cannot be clearly established by any investigation. Although therapy that aims to correct the underlying cause is rational, for many patients, such an approach is not possible. Few randomized trials have been conducted in this setting, and the sample sizes in the trials that have been conducted are small. The use of a corticosteroid or octreotide for bowel obstruction is supported by randomized trials. The 5-HT_3_ receptor antagonists have not been established to be efficacious for nausea resulting from opioid administration, but they may be helpful for nausea of uncertain origin in patients with advanced cancer. Given the limited data from clinical trials, a sequential pharmacologic approach based largely on past practise and considerations of costs and adverse effects is reasonable.

Chemotherapy-related nausea and vomiting remains a problem in many patients despite the use of 5-HT_3_ receptor antagonists and dexamethasone. As an add-on to standard therapy, the NK_1_ receptor antagonist aprepitant reduces the likelihood of vomiting or retching in association with cisplatin or anthracycline-plus-cyclophosphamide chemotherapy by an absolute 15%–20%. Add-on aprepitant is recommended as first-line therapy by several prominent guidelines groups. Olanzapine and cannabinoids have been suggested as potentially useful interventions, but data from phase iii clinical trials are lacking.

## Figures and Tables

**TABLE I:** Causes of nausea and vomiting in the patient with cancer

Chemotherapy
Radiation therapy, especially to upper abdomen
Other medications (for example, opioids)
Cancer
Metabolic effects (for example, hypercalcemia, hyponatremia)
Impaired gastric emptying (for example, ascites)
Gastrointestinal obstruction
Central nervous system metastases (for example, brain)
Others (for example, anxiety, severe pain)

**TABLE II:** Correctable causes of cancer-related emesis

Cause	Specific therapy a
Brain metastases	Radiation therapy
Hypercalcemia	Bisphosphonates
Hyponatremia (siadh)	Demeclocyline
Ascites	Paracentesis
Medication	Substitution, discontinuation or reintroduction (in the case of withdrawal reaction) of the responsible drug
Infection	Antibiotic therapy
Gastritis	Discontinue irritant drug or add a proton pump inhibitor
Bowel obstruction	Gastric venting tube, disimpaction

a In most instances, effective treatment of the underlying cancer is helpful as well.

siadh = syndrome of inappropriate secretion of antidiuretic hormone.

**TABLE III:** Drugs with antiemetic activity

Drug class	Example
Phenothiazines/butyrophenones	Prochlorperazine
Substituted benzamide	Metoclopramide
Antihistamines	Dimenhydrinate
Corticosteroid	Dexamethasone
Anticholinergic	Scopolamine
Cannabinoid	Nabilone
5-HT_3_ receptor antagonist	Granisetron
Benzodiazepine	Lorazepam
NK_1_receptor antagonist	Aprepitant

5-HT_3_ = 5-hydroxytryptamine3; NK_1_ = neurokinin 1.

**TABLE IV:** Phase III results with aprepitant

Reference	Patients (*n*)	Chemotherapy type	Result
Hesketh *et al.,* 2003 19	520	Cisplatin > 70 mg/m^2^	Superior cr with aprepitant (72.7% vs. 52.3%, *p<*0.01); 22.7% difference in no-vomiting rate.
Poli-Bigelli *et al.,* 2003^20^	523	Cisplatin > 70 mg/m^2^	Superior cr with aprepitant (62.7% vs. 43.3%, *p<*0.001); 22% difference in no-vomiting rate.
Warr *et al.,* 2005^10^	857	Anthracycline plus cyclophosphamide	Superior cr with aprepitant (50.8% vs. 42.5%, *p=*0.015); 17% difference in no vomiting rate.
Schmoll *et al.,* 2006^21^	489	Cisplatin >70 mg/m^2^	Superior cr with aprepitant (72% vs. 61%, *p*=0.003); 14.3% difference in no-vomiting rate.

cr = complete response (defined as no vomiting or retching, and no use of “as needed” antiemetics from hour 0 to hour 120).

**TABLE V:** First-line antiemetic therapy for chemotherapy

Chemotherapy	Antiemetic combination
Cisplatin > 50 mg/m^2^	Dexamethasone 12 mg orally day 1, 8 mg orally daily days 2–4 Granisetron 2 mg orally (1 mg intravenously) OR ondansetron 24 mg orally (8 mg intravenously) OR dolasetron 100 mg orally (or intravenously) OR palonosetron 0.25 mg intravenously day 1 Aprepitant 125 mg orally day 1, 80 mg orally days 2–3
Anthracycline plus cyclophosphamide	Dexamethasone 12 mg orally day 1, 4 mg orally daily days 2–3 Granisetron 2 mg orally (1 mg intravenously) OR ondansetron 8 mg orally twice daily (8 mg intravenously) OR dolasetron 100 mg orally (or intravenously) OR palonosetron 0.25 mg intravenously day 1 Aprepitant 125 mg orally day 1, 80 mg orally days 2–3
Carboplatin, irinotecan, low-dose cisplatin	Dexamethasone 10 mg intravenously, 4 mg orally twice daily days 2–3 (for low-dose cisplatin: 10 mg intravenously for at least 3 days) Granisetron 2 mg orally (1 mg intravenously) OR ondansetron 8 mg orally twice daily (8 mg intravenously) OR dolasetron 100 mg orally (or intravenously) OR palonosetron 0.25 mg intravenously day 1

**TABLE VI:** Approach when first-line therapy fails

Chemotherapy	Antiemetic combination
Cisplatin > 50 mg/m^2^	Consider adding olanzapine 5 mg orally daily OR nabilone 0.5 mg twice daily OR dronabinol 5 mg three times daily for the days of nausea or vomiting
Anthracycline plus cyclophosphamide	Same as for cisplatin
Carboplatin, irinotecan, low-dose cisplatin	Consider adding aprepitant 125 mg orally day 1 and 80 mg orally on days 2–3
